# Doppler Ultrasonography for Assessing Uterine Viability and Fetal Vitality in Uterine Torsion in a Domestic Cat (*Felis catus*): A Case Report

**DOI:** 10.3390/ani16172806

**Published:** 2026-09-07

**Authors:** Catalin Micsa, Maria Roxana Turcu, Ioana-Raluca Prunean, Alexandru Diaconescu

**Affiliations:** Department of Clinical Sciences II, Faculty of Veterinary Medicine, University of Agronomic Sciences and Veterinary Medicine of Bucharest, 59 Marasti Blvd, District 1, 011464 Bucharest, Romania

**Keywords:** queen, feline, uterine torsion, Doppler ultrasonography, resistive index, pulsatility index, theriogenology, ovariohysterectomy

## Abstract

Background: Uterine torsion is rare in domestic cats and, when diagnosis is delayed, is associated with high maternal and fetal mortality. Conventional B-mode ultrasonography cannot reliably distinguish viable from devitalised uterine tissue, and Doppler ultrasonography, despite its capacity to assess uterine, ovarian, and fetal perfusion, has not been systematically applied to feline uterine torsion. Case Description: A 4-year-old queen in late gestation presented with acute abdominal pain, lethargy, abdominal distension, and tachypnoea. B-mode imaging showed an enlarged, fluid-distended uterine segment with thickened walls. Colour, spectral, and power Doppler revealed an absent to markedly reduced arterial flow distal to the torsion, elevated resistive and pulsatility indices in the uterine and ovarian arteries, and the loss of peripheral perfusion. The absence of fetal heartbeats and umbilical flow confirmed fetal nonviability. Laparotomy confirmed the torsion of a congested, devitalised uterine horn, with asymmetric fetal loading (two kittens in the torted horn, one fetal resorption in the contralateral horn) as the likely biomechanical cause. Ovariohysterectomy was performed without derotation. Histopathology confirmed haemorrhagic necrosis, and haematology showed severe regenerative anaemia and marked leucocytosis, which resolved progressively after surgery. Conclusions: Doppler ultrasonography supplied vascular and fetal viability information unavailable from B-mode imaging alone, supporting ovariohysterectomy over conservative detorsion. Based on the feline case reports identified during the preparation of this report, this appears to be among the first descriptions of a systematic, multi-vessel Doppler protocol combining colour, power, and spectral modalities with resistive and pulsatility index measurements in feline uterine torsion; as this observation derives from a single case, it should be regarded as preliminary.

## Simple Summary

Uterine torsion, characterised by a twisting of the uterus that obstructs its blood supply, is a rare but potentially fatal condition in cats, particularly in pregnant queens nearing the end of gestation. This report details a case of a pregnant cat presenting with acute abdominal pain, restlessness, and distress. The veterinary team initially used B-mode ultrasound to assess the abdomen, revealing an enlarged, fluid-filled uterus. However, this modality alone could not determine tissue viability. Doppler ultrasound was subsequently used to evaluate the blood flow within the uterine and fetal vessels. The examination indicated a markedly reduced blood flow to the affected uterine region and the absence of detectable fetal heartbeats or umbilical flow. This critical information enabled the surgical team to make a prompt decision to perform an ovariohysterectomy rather than attempt preservation. The cat recovered successfully. This case suggests that Doppler assessment may add useful information to the emergency evaluation of acutely ill gravid queens, although further cases are needed to confirm its clinical value.

## 1. Introduction

Uterine torsion is the rotation of a uterine horn or the uterine body about its longitudinal axis [1,2]. The twisting compresses the uterine vasculature within the mesometrium and broad ligament, progressively occluding the venous outflow and, in more severe or prolonged cases, the arterial inflow [1,2]. The resulting ischaemia causes vascular congestion, uterine wall oedema, and haemorrhagic necrosis, culminating in maternal and fetal death if intervention is delayed [3,4,5]. The degree of torsion ranges from 90° to 900° [1,2], and both unilateral and bilateral uterine horn involvement have been described.

In domestic cats (*Felis catus*), uterine torsion is substantially less common than in cattle and buffaloes, where it is a principal cause of prepartum dystocia [1,4,5]. The published feline literature comprises only individual case reports [1,2,3,4,5,6,7], reflecting both the rarity of the condition and the diagnostic difficulty it poses. Most reported feline cases have occurred during mid- to late gestation, and, in most, the diagnosis was confirmed only at exploratory laparotomy [1,2,3,4,5,6,7]. Proposed predisposing factors include the laxity of the broad ligament resulting from previous pregnancies, excessive fetal mobility, uterine wall hypotonia, vigorous physical activity, and concurrent uterine pathology such as pyometra, hydrometra, or mucometra [2,3,7,8,9].

A biomechanical predisposing factor that has received limited attention in the feline literature is asymmetric intraluminal fetal loading. The feline uterus is bicornuate, with two relatively mobile horns suspended from the dorsal abdominal wall by the broad ligament. When fetal number and size are unequally distributed between the two horns, the heavier horn generates a greater gravitational torque and imposes differential tension on the mesometrial attachments, producing the mechanical asymmetry that can initiate and sustain torsion [1,2,3]. In the present case, an asymmetric fetal distribution was observed intraoperatively: two kittens occupied one uterine horn, while the contralateral horn showed evidence of fetal resorption. This distribution is compatible with, but does not by itself establish, a mechanical contribution to torsion of the more heavily loaded horn; causality cannot be inferred from a single observation. Whether asymmetric fetal distribution, detectable ultrasonographically in the antepartum period, represents a genuine risk factor for feline uterine torsion warrants investigation in a larger case series.

Clinical signs are nonspecific and vary with the degree and duration of torsion, as well as the gestational stage. Affected queens commonly exhibit lethargy, anorexia, acute abdominal pain, abdominal distension, tachypnoea, and haemorrhagic vulvar discharge [1,2,3,4,5,6,7]. These findings overlap substantially with those of pyometra, hydrometra, and other acute abdominal conditions, complicating pre-surgical diagnosis [5,6,8]. A physical examination may reveal pale or congested mucous membranes, tachycardia, and haemodynamic compromise due to haemorrhagic sequestration into the torsion-affected horn [3,4]. Laboratory evaluation frequently reveals severe regenerative anaemia, leucocytosis with a left-shifted neutrophilia, and, in severe cases, electrolyte disturbances and coagulopathic abnormalities consistent with disseminated intravascular coagulation [3].

Conventional diagnostic imaging—including plain radiography and B-mode ultrasonography—contributes to the assessment of the case by identifying uterine distension, free peritoneal fluid, and fetal number. However, B-mode ultrasonography alone cannot reliably differentiate viable from devitalised uterine tissue [4,5]. The greyscale appearance of a fluid-distended, thick-walled uterine segment is shared by multiple pathological entities, and the detection of fetal cardiac silence on real-time B-mode imaging, whilst consistent with fetal death, does not quantify the degree of vascular compromise or guide the surgical decision between detorsion and ovariohysterectomy.

Doppler ultrasonography, encompassing colour flow Doppler, power Doppler, and spectral (pulsed-wave) Doppler modalities, provides a non-invasive method for evaluating tissue perfusion at the vessel level. Spectral Doppler waveform analysis allows the calculation of the resistive index (RI = [peak systolic velocity (PSV) − end-diastolic velocity (EDV)]/PSV) and the pulsatility index (PI = [PSV − EDV]/time-averaged maximum velocity), both of which reflect downstream vascular resistance [10,11]. Elevated RI and PI values indicate an increased impedance to flow, consistent with vascular compression or obstruction [10,11]. In ruminants, the Doppler interrogation of the uterine artery reliably distinguishes torsion-affected from normally perfused tissue, with a significantly elevated PI and RI ipsilateral to the torsion site and marked attenuation or abolition of end-diastolic flow [10,11,12,13]. In small companion animals, the Doppler assessment of uterine arterial haemodynamics has been described during normal canine parturition and the puerperium [12], but published reference data for normal feline uterine and ovarian artery Doppler indices throughout gestation remain sparse. The application of Doppler ultrasonography in the specific context of feline uterine torsion has been mentioned only incidentally in a small number of recent case reports [5,7], and no published case has described a systematic, multi-vessel Doppler protocol addressing uterine, ovarian, and umbilical artery flow indices and their clinical interpretation in this species.

This case report describes the use of colour, spectral, and power Doppler ultrasonography as an adjunct to B-mode imaging in the emergency evaluation of a 4-year-old gravid queen presenting with acute abdominal signs attributable to uterine torsion. The aims are to document the Doppler vascular findings in detail, to demonstrate the value of this modality in distinguishing viable from devitalised tissue and in determining fetal vitality, and to illustrate how a multi-vessel Doppler assessment informed the decision to perform ovariohysterectomy rather than conservative detorsion.

## 2. Materials and Methods

### 2.1. Case Presentation

A 4-year-old intact female domestic cat (*Felis catus*), known as Lia, presented on an emergency basis to the USAMV Bucharest Veterinary Teaching Hospital on 23 June 2026 at 02:33. Lia was a previously stray queen; a member of the public who had observed her within the local community came forward at admission and subsequently assumed responsibility as her owner. The reported 6–8-h history of acute abdominal pain, progressive lethargy, abdominal distension, and tachypnea, with no progression of labour despite signs of discomfort, and the reproductive history of two prior litters (litter sizes of three and four kittens, with no reported complications), were provided by this individual based on prior observation of the queen.

On physical examination, the queen was lethargic and in visible distress. Vital parameters were as follows: rectal temperature, 37.1 °C; heart rate, 180 bpm; respiratory rate, 40 breaths/min. Mucous membranes were pale with a capillary refill time of 2–3 s. Abdominal palpation revealed marked diffuse distension and pain concentrated in the mid-to-caudal quadrant (Figure S5). No fetal parts were accessible per vaginam on digital examination. The queen was classified as a haemodynamically unstable emergency patient. Intravenous access was secured via cephalic vein catheterisation, and isotonic crystalloid fluid therapy was initiated as a 10 mL/kg intravenous bolus over 15 min, with reassessment of perfusion parameters before further boluses to support haemodynamic stability during the diagnostic work-up.

### 2.2. Laboratory Analyses

Venous blood was collected from the cephalic vein into dipotassium ethylenediaminetetraacetate (K2EDTA)-anticoagulated tubes for complete blood count (CBC) and reticulocyte analysis. Serial haematological evaluations were performed at three time points: on admission (23 June 2026, 02:33; Sample No. 4803), the following day before surgery (24 June 2026, 13:42; Sample No. 4808), and the first post-operative morning (25 June 2026, 10:52; Sample No. 4813). All analyses were performed on the Mindray BC-60R Vet haematology analyser (Mindray Animal Care, Shenzhen, China) at the USAMV București laboratory. Reference intervals were those of the analyser manufacturer for the domestic cat. Complete results are presented in Table 1.

### 2.3. B-Mode Ultrasonographic Examination

The ventral and lateral abdomen was clipped of hair and liberally coated with acoustic coupling gel. The queen was positioned initially in dorsal recumbency, with repositioning to lateral recumbency as required. B-mode abdominal ultrasonography was performed using a Mindray Animal Care Vetus 9 (Mindray Animal Care, Shenzhen, China) with a microconvex transducer operating at 5 to 10 MHz. Both uterine horns and the uterine body were systematically interrogated from the pubic brim cranially to the level of the ovaries bilaterally. Parameters recorded included the following: uterine horn diameter and wall thickness, echogenicity and nature of intraluminal fluid or solid contents, serosal surface continuity, volume and character of any free peritoneal effusion, fetal number and topographic distribution within each horn, fetal biparietal diameter and crown-rump length where measurable, and real-time fetal cardiac activity. Fetal cardiac absence was defined as no discernible rhythmic myocardial motion during a minimum 30 s continuous observation period.

### 2.4. Doppler Ultrasonographic Examination

Doppler ultrasonographic examination was performed immediately after B-mode assessment, using the same Mindray Vetus 9 system fitted with a C7-3s microconvex transducer operating at 5 to 7 MHz. Colour flow Doppler was used to map vascular signal within the uterine wall proximal and distal to the torsion site in both horns, within the ovarian pedicles bilaterally, and within the fetal umbilical regions. Power Doppler was applied to assess peripheral uterine wall perfusion and placental vascular distribution, given its greater sensitivity to low-velocity flow.

Spectral (pulsed-wave) Doppler measurements were obtained at the ipsilateral and contralateral uterine arteries and the ipsilateral ovarian artery, at sites where colour Doppler had first confirmed a detectable vascular signal. Instrument gain, time-gain compensation, and the colour/power Doppler velocity scale were held constant across all interrogated vessels to permit direct qualitative comparison between the affected and contralateral sides. The sample volume gate was set to 1.5 mm and the pulse repetition frequency to 1.0 kHz, with a wall filter of 50 Hz. The transducer was positioned to keep the angle of insonation below 60 degrees under continuous real-time colour Doppler guidance. Three consecutive waveforms were assessed at each site. The resistive index (RI = [PSV − EDV]/PSV) and pulsatility index (PI = [PSV − EDV]/time-averaged maximum velocity) were calculated in real time by the system’s built-in software and used to guide interpretation of flow impedance during the examination; numeric values displayed by the system were not exported or archived, and Doppler findings are therefore reported qualitatively rather than as absolute index values (see Section 4.7, Limitations).

### 2.5. Surgical Intervention

As Lia had no previously registered owner, confirming the caretaker’s assumption of ownership and obtaining written informed consent for surgical intervention required a period of arrangement following admission; during this interval, the queen was maintained on intravenous fluid therapy and antibiotherapy (Ceftriaxone 25 mg/kg twice daily) with serial haemodynamic and haematological monitoring (Table 1). Once consent was confirmed, the queen was prepared for emergency surgery on 24 June 2026. Premedication consisted of a combination of Dexmedetomidine, Butorphanol, and Ketamine administered intramuscularly at doses of 3 mcg/kg, 0.3 mg/kg, and 2 mg/kg, respectively. Induction was achieved with Propofol administered intravenously to effect a dose of 2 mg/kg. Following orotracheal intubation with a 3 mm cuffed endotracheal tube, anaesthesia was maintained with Isoflurane in 100% oxygen delivered via a non-rebreather circuit. Monitoring included heart rate, respiratory rate, peripheral oxygen saturation (SpO_2_), end-tidal carbon dioxide concentration (ETCO_2_), and non-invasive (oscillometric) blood pressure throughout the procedure.

Due to the septic state, the patient became hypotensive during surgery. As treatment, we administered a colloid fluid bolus to increase the mean arterial blood pressure to 60 mmHg or higher.

A midline ventral celiotomy was performed from the umbilicus to the pubis. On entering the abdominal cavity, serosanguineous peritoneal fluid was noted (approximately 50 mL). The right uterine horn was markedly enlarged, dark red to purple in colour, and congested with evidence of haemorrhagic mural oedema. A 540-degree torsion about the longitudinal axis was confirmed at the base and ovarian ends, with clockwise rotation. The broad ligament on the affected side was partially torn. The contralateral uterine horn appeared mildly congested with an intact vascular supply.

Without prior manual derotation—a decision supported by the pre-operative Doppler evidence of complete vascular compromise in the affected horn and by published evidence that reperfusion of a devitalised uterus risks systemic release of endotoxins and inflammatory mediators [3,5,7]—an ovariohysterectomy was performed by sequential ligation of the ovarian pedicles and the uterine body at the level of the cervix, followed by en-bloc resection. The abdominal cavity was lavaged with warm saline. Routine layered closure was performed.

### 2.6. Post-Operative Care

Post-operative analgesic and antimicrobial therapy were administered as follows: Buprenorphine 20 mcg/kg IV was given at 8 h, and antimicrobial treatment was completed with Metronidazole 11 mg/kg IV every 12 h until discharge. Fluid therapy was continued post-operatively until voluntary water and food intake resumed. The queen was monitored in hospital until stable and discharged on post-operative day 3.

### 2.7. Ethical Statement

This case report describes a veterinary clinical case in which all procedures were performed solely in the patient’s best interests as standard emergency care.

Written informed consent for clinical management and publication of case data was obtained from the individual who assumed ownership of the previously stray queen.

## 3. Results

### 3.1. Haematological Findings

Sequential haematological assessment at three time-points revealed a severe, markedly regenerative anaemia with persistent leucocytosis throughout the perioperative period (Table 1).

On admission (23 June 2026, 02:33), the erythrocyte count was 1.86 × 10^12^/L (reference interval [RI]: 6.30–11.82 × 10^12^/L), haemoglobin 29 g/L (RI: 90–160 g/L), and haematocrit 0.088 L/L (RI: 0.260–0.502 L/L), consistent with severe anaemia. The reticulocyte count was markedly elevated at 140.2 × 10^9^/L (RI: 4.0–52.0 × 10^9^/L), with an immature reticulocyte fraction (IRF) of 56.8% (RI: 0.0–33.0%), a mid-fluorescence reticulocyte fraction (MFR) of 29.8% (RI: 0.0–25.8%), and a high-fluorescence reticulocyte fraction (HFR) of 27.0% (RI: 0.0–8.5%). The reticulocyte pool was predominantly composed of high- and mid-fluorescence subtypes. The total leucocyte count on admission was 83.78 × 10^9^/L (RI: 3.46–17.50 × 10^9^/L), driven predominantly by severe absolute neutrophilia (64.51 × 10^9^/L; RI: 1.95–11.50 × 10^9^/L); a suspected band neutrophil population was flagged on the analyser scattergram, indicating a left shift. Concurrent lymphocytosis (12.73 × 10^9^/L), monocytosis (4.11 × 10^9^/L), eosinophilia (1.76 × 10^9^/L), and basophilia (0.67 × 10^9^/L) were observed. The platelet count was within the reference interval at 256 × 10^9^/L.

Pre-operatively on 24 June 2026 (13:42), the erythrocyte parameters showed marginal change (RBC 1.76 × 10^12^/L; HGB 31 g/L; HCT 0.092 L/L), while the total leucocyte count had declined to 58.49 × 10^9^/L (neutrophils 42.81 × 10^9^/L). The reticulocyte count had increased slightly to 152.9 × 10^9^/L with an IRF of 66.7%. The platelet count was 153 × 10^9^/L but was flagged for platelet clumping (PLT Clump); the true thrombocyte count may have been higher.

On the first post-operative morning (25 June 2026, 10:52), the erythrocyte parameters showed partial recovery (RBC 2.24 × 10^12^/L; HGB 37 g/L; HCT 0.125 L/L). The total leucocyte count continued to decline (45.26 × 10^9^/L; neutrophils 28.38 × 10^9^/L), although all leucocyte parameters remained above the reference interval. The platelet count rose to 412 × 10^9^/L. The reticulocyte count remained elevated (164.2 × 10^9^/L; IRF 58.8%; MFR 35.0%; HFR 23.8%), confirming a sustained regenerative response. The immature platelet fraction (IPF) was 15.5%, indicating concurrent thrombopoietic stimulation. RDW-CV was markedly elevated at all three time-points (0.623, 0.598, 0.591; RI: 0.142–0.266), reflecting a marked erythrocyte size heterogeneity from the mixture of mature red cells and newly released larger reticulocytes.

### 3.2. B-Mode Ultrasonographic Findings

**Figure 1:**
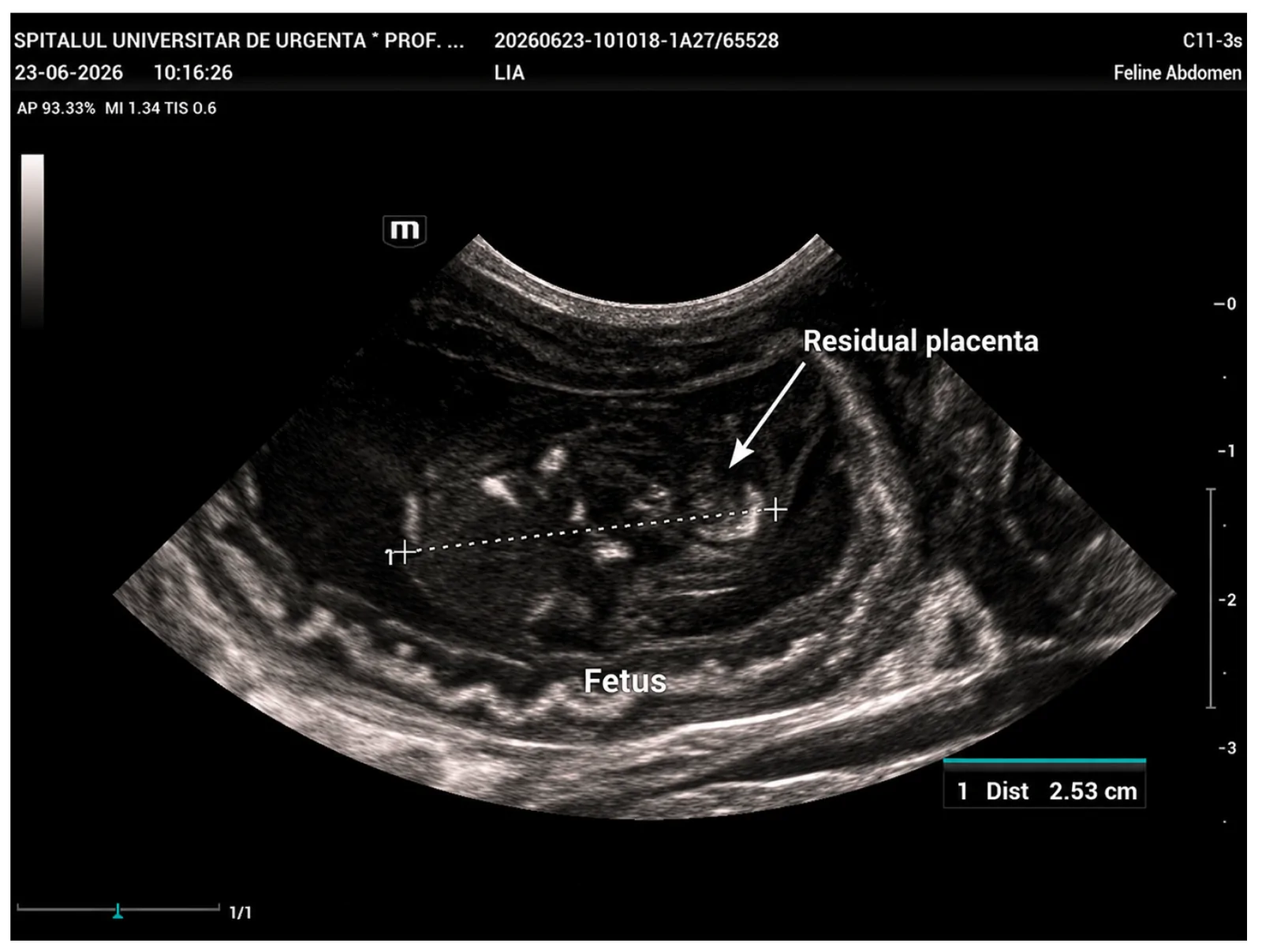
B-mode ultrasonographic image of a foetus within the torted uterine horn, with caliper measurement of crown-rump length (2.53 cm). No fetal cardiac activity was detected on real-time observation.

**Figure 2:**
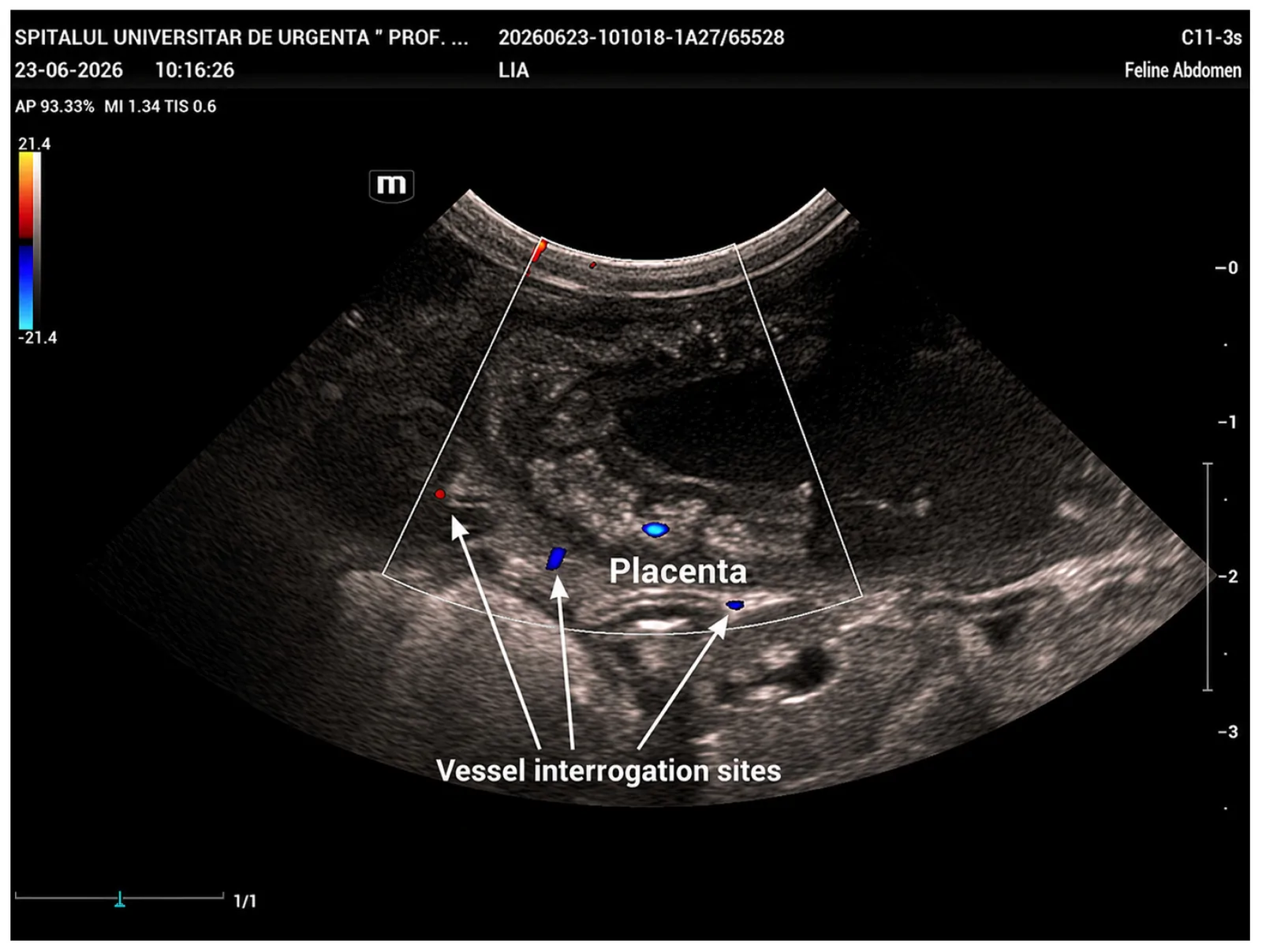
Colour Doppler ultrasonographic image of the contralateral uterine horn, showing a residual placental mass (“Placenta”) without an identifiable foetus or fetal heartbeat, and only minimal scattered vascular signal, consistent with fetal resorption.

The B-mode ultrasonographic examination identified an enlarged, markedly distended uterine segment in the mid-to-caudal abdomen. The affected uterine horn exhibited markedly thickened, hyperechoic walls and a distended lumen containing anechoic to hypoechoic fluid and heterogeneous solid contents consistent with fetal tissue. The contralateral horn was less distended. Two distinct fetal structures were identified within the affected horn. Real-time B-mode imaging demonstrated the absence of fetal cardiac activity in both foetuses during a minimum 30 s continuous observation period at each site (Figure 1). A residual placental mass without an identifiable foetus, consistent with earlier resorption, was noted in the contralateral horn (see Section 3.3, Figure 2). B-mode imaging alone did not permit a reliable assessment of the degree of uterine wall ischaemia or vascular compromise.

### 3.3. Doppler Ultrasonographic Findings

The colour flow Doppler mapping of the right uterine horn demonstrated markedly reduced or absent arterial colour signals within the uterine wall distal to the torsion site, with turbulent or chaotic flow patterns at the site of maximum torsion. The contralateral uterine artery and horn showed partially preserved colour flow signals, with the residual placental mass corresponding to the site of fetal resorption (Figure 2).

The colour Doppler interrogation of the residual placental mass in the contralateral horn, performed using the same Mindray Vetus 9 system and C7-3s microconvex transducer (5–7 MHz) used throughout the examination, demonstrated only a minimal, scattered vascular signal, distinct from the near-complete absence of colour flow distal to the torsion site in the affected horn. Formal spectral waveform quantification (RI and PI) was not performed on this vessel. This qualitative asymmetry in the colour Doppler signal between the affected and unaffected sides is consistent with the pattern of ipsilateral perfusion loss reported in caprine and bovine uterine torsion [11].

Ovarian artery interrogation on the affected side revealed markedly elevated-resistance waveform Doppler signals, consistent with vascular compromise at the level of the ovarian pedicle. Umbilical cord vascular flow was absent for both foetuses in the torted horn; no vascular signal was identifiable within the residual placental mass in the contralateral horn, confirming fetal cardiovascular arrest. No fetal cardiac activity was detected on combined B-mode and colour Doppler assessment in either kitten (Table 2).

### 3.4. Intraoperative Findings

**Figure 3:**
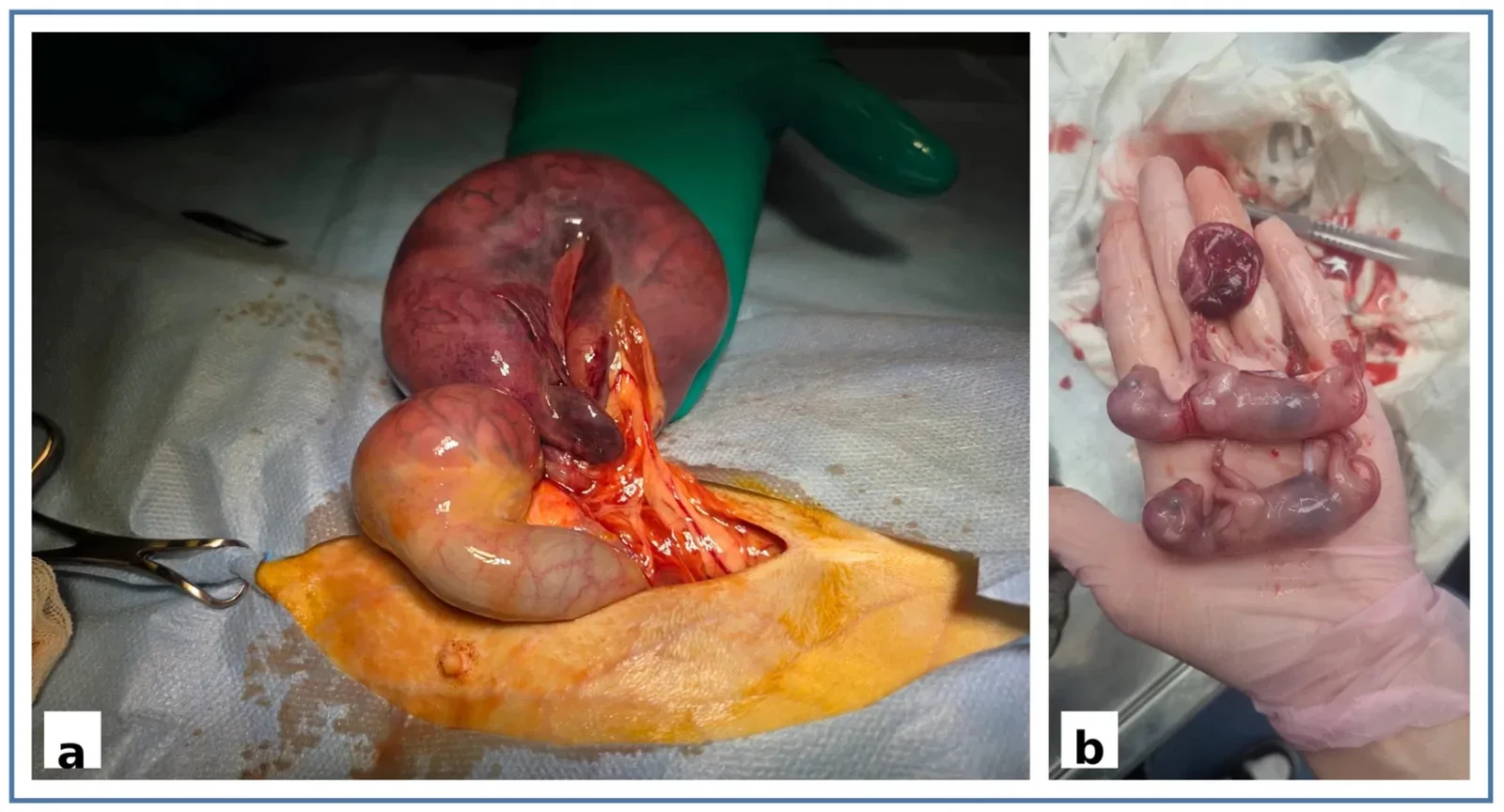
Intraoperative findings: (**a**) exteriorised uterus showing the torted horn markedly enlarged, dark red to purple, with haemorrhagic mural oedema and serosal discolouration; and (**b**) the two kittens from the torted horn, alongside fetal resorption in the contralateral horn, illustrate the asymmetric intraluminal mass distribution between the two horns.

During exploratory laparotomy, approximately 50 mL of serosanguineous peritoneal fluid was noted. The right uterine horn was markedly enlarged, dark red to purple, and congested, with haemorrhagic mural oedema and serosal discolouration. A 540-degree clockwise torsion about the longitudinal axis was confirmed at the base and ovarian end (Figure 3a, Video S1, Figures S1–S3).

The broad ligament on the affected side showed partial tearing with clotted blood, and the ovarian vessels were engorged, with evidence of ischaemic necrosis. The contralateral horn was mildly congested, with an intact vascular supply.

The fetal distribution was asymmetric: two kittens were present in the torted horn, and evidence of fetal resorption was observed in the contralateral horn (Figure 3b). Both kittens were nonviable. The mass disparity between the two horns was considered the biomechanical substrate for torsion in the more heavily loaded horn. This pattern of asymmetric fetal loading is discussed in Section 4.

Ovariohysterectomy was performed without prior manual derotation, using the sequential ligation of the ovarian pedicles and uterine body, followed by en-bloc resection. The abdominal cavity was lavaged with warm saline, and the incision was closed routinely.

### 3.5. Histopathological Examination

A histopathological examination of the excised specimen confirmed the haemorrhagic necrosis of the uterine wall in the torted horn. Microscopic features included transmural haemorrhage, intravascular thrombosis, and full-thickness necrosis of the endometrium, myometrium, and serosa, consistent with ischaemia secondary to complete vascular occlusion. The contralateral horn showed advanced resorption with minimal residual tissue. No evidence of pre-existing uterine pathology was identified in either horn.

### 3.6. Post-Operative Course

The queen recovered from general anaesthesia without complications. A post-operative haematological assessment on 25 June 2026 (Table 1) showed the partial recovery of erythrocyte indices (RBC 2.24 × 10^12^/L; HGB 37 g/L; HCT 0.125 L/L), a declining but still markedly elevated neutrophil count (28.38 × 10^9^/L), reactive thrombocytosis (PLT 412 × 10^9^/L), and a sustained active regenerative response (RET# 164.2 × 10^9^/L; IRF 58.8%). The queen resumed the voluntary intake of food and water within 6 h post-operatively and was discharged from hospital on post-operative day 3 (Figure S4). No complications were recorded during hospitalisation, at discharge, or at subsequent follow-up.

## 4. Discussion

This case describes the emergency management of uterine torsion in a 4-year-old gravid queen presenting with acute abdominal signs, severe regenerative anaemia, and marked leucocytosis. Ovariohysterectomy was performed without prior derotation following a systematic pre-operative Doppler ultrasonographic assessment that confirmed complete vascular compromise in the affected uterine horn and the absence of fetal cardiovascular activity. The queen recovered fully. Two findings warrant specific discussion: the systematic Doppler vascular characterisation and the intraoperative confirmation of asymmetric fetal loading as the probable biomechanical predisposing factor.

### 4.1. Rarity and Clinical Context

Uterine torsion in the domestic cat is substantially less common than in cattle, buffaloes, and mares, where it constitutes a principal or frequent cause of prepartum dystocia [1]. The exact prevalence in cats is not known, and the published literature consists exclusively of individual case reports [1,2,3,4,5,6,7,14] (Table 3). We have also previously reported on the related condition of uterine prolapse in cats [15], which shares some predisposing anatomical features with torsion but is a distinct clinical entity. The true incidence of uterine torsion specifically is likely underestimated, since affected outdoor and unowned cats may die or be euthanised without a definitive diagnosis or necropsy, and such cases would never enter the published literature.

Most cases involved gravid queens in mid- to late gestation, although uterine torsion has also been described in non-gravid queens with pyometra [8] and in the absence of concurrent uterine pathology [9]. The present case is consistent with the established clinical profile: late gestation, acute onset, nonspecific systemic signs, and a diagnosis confirmed at surgery.

### 4.2. Haematological Findings in Comparative Context

The haematological profile in this case—haematocrit 0.088 L/L at presentation, markedly elevated reticulocytes with high IRF, MFR, and HFR, and leucocytosis of 83.78 × 10^9^/L with severe neutrophilia—is consistent with, and, in some parameters, more severe than, the haematological findings reported in previously published cases of feline uterine torsion. Ridyard et al. (2000) reported marked anaemia, leucocytosis, mild thrombocytopenia, and suspected DIC, as evidenced by a prolonged activated partial thromboplastin time, in a 15-month-old Maine Coon with a full-term pregnancy (7 kittens) [3]. Ali et al. (2021) documented mild anaemia (haematocrit 22%, haemoglobin 7 g/dL), leucocytosis (19.5 × 10^3^/uL), and hypoglycaemia in a 2-year-old queen with a 720-degree right horn torsion [14].

The present case differs from both in the degree of erythropoietic stimulation: the reticulocyte count of 140.2–164.2 × 10^9^/L across three time-points, together with IRF values of 56.8–66.7% (normal ≤ 33%), MFR of 29.8–36.3% (normal ≤ 25.8%), and HFR of 23.8–30.4% (normal ≤ 8.5%), indicates an intense acute marrow response to haemorrhagic sequestration. Moreover, blood transfusion was not performed due to the unavailability of a blood product, and the patient’s clinical status was considered stable, with ongoing reticulocytosis.

As in ruminants, where uterine-torsion-associated anaemia is attributed to haemorrhagic accumulation within the congested uterine wall, the two kittens retained within the torted horn in this case likely increased the pooled blood volume, plausibly contributing to the severity of anaemia observed here.

The progressive decline in total leucocyte and neutrophil counts confirms that the inflammatory leucogram was driven by necrotic uterine tissue, resolving once it was removed; post-operative reactive thrombocytosis is a similarly expected response to the removal of an acute inflammatory and septic focus, and all parameters remained above the reference interval only as far as the immediate post-surgical tempo of normalisation would predict. Unlike the Ridyard et al. (2000) case, in which a prolonged aPTT raised the suspicion of DIC [3], no coagulopathy was demonstrated here, which may reflect a shorter ischaemic interval, less endothelial and tissue-factor exposure, or simply the absence of dedicated coagulation testing.

### 4.3. Asymmetric Fetal Loading as a Biomechanical Substrate

The intraoperative confirmation of two kittens in the torted horn and fetal resorption in the contralateral horn is consistent with the hypothesis that asymmetric intraluminal mass distribution contributes mechanically to feline uterine torsion, although causality cannot be established from a single case. The feline bicornuate uterus is suspended bilaterally from the dorsal abdominal wall by the broad ligament. In late gestation, as the ligament progressively stretches, an unequal fetal mass between the two horns creates asymmetric gravitational loading and differential mesometrial tension. If the ligament’s viscoelastic resistance is insufficient to counteract this torque—particularly during vigorous fetal movement or maternal locomotion—torsion can be initiated and sustained.

A strikingly similar fetal distribution was described by Ali et al. (2021) in their case of 720-degree right horn torsion: two kittens were recovered from the torted right horn and one from the contralateral left horn [14]. The identical 2 + 1 distribution in two independently reported cases is suggestive, and the consistent pattern is compatible with the hypothesis that the two-kitten horn becomes the pivot of the torsion. The systematic antepartum ultrasonographic documentation of the fetal number per horn and relative horn diameter could identify queens at an elevated biomechanical risk. No published feline case report has performed this prospective ante-mortem risk stratification, and it represents a practical direction for future study.

### 4.4. Doppler Ultrasonography: Novel Contribution and Comparative Data

The primary contribution of this report is the systematic, multi-vessel Doppler characterisation of uterine torsion in a cat, combining colour, power, and spectral (qualitative) assessment across the uterine, ovarian, and umbilical circulations. Previous publications have mentioned a Doppler assessment incidentally [5,7] but have not described the modality-specific findings, or the vessels interrogated, the technical parameters, or the calculated resistance indices.

Colour Doppler demonstrated absent to markedly reduced arterial signals distal to the torsion, and power Doppler confirmed the near-total loss of peripheral mural perfusion. The within-patient Doppler asymmetry in the present case was unambiguous and independently supportable without reference to external normative data.

The fetal cardiovascular assessment by umbilical cord Doppler and combined B-mode/colour Doppler revealed a complete absence of umbilical flow and cardiac activity in both kittens. This finding, combined with the uterine artery data, provided a pre-operative assessment that the affected horn and both foetuses were nonviable, precluding any surgical strategy aimed at fetal salvage and supporting the decision to proceed immediately with ovariohysterectomy.

### 4.5. Surgical Decision: Ovariohysterectomy Without Derotation

The decision to perform en-bloc ovariohysterectomy without prior manual detorsion was based on three converging lines of evidence: the Doppler confirmation of complete vascular occlusion in the affected horn, B-mode evidence of fetal death, and the published consensus that reperfusion of an ischaemic uterine segment risks releasing inflammatory mediators, endotoxins, and reactive oxygen species into the systemic circulation, potentially precipitating cardiovascular collapse or DIC [3,5,7]. This surgical approach is consistently advocated in the published literature on feline and ruminant surgery.

In the present case, the pre-operative Doppler findings provided an objective, pre-surgical confirmation of the rationale for immediate OHE, rather than relying solely on intraoperative judgement.

### 4.6. Comparison with Published Cases

A comparison of the present case with all published reports of feline uterine torsion is presented in Table 3. The torsion degree in previously reported cases has ranged from 90 degrees [6] to 720 degrees [14], predominantly involving the left horn [2,6,7] when the affected side was documented. All reported queens survived following ovariohysterectomy. Fetal survival was reported only in the Kuroda et al. (2017) case, where one kitten from the contralateral horn was alive at the time of laparotomy but died on post-operative day 1 [4]. In the present case, all kittens were nonviable, confirmed by the combined B-mode and Doppler assessment before laparotomy.

### 4.7. Limitations

This report is subject to the limitations inherent to a single case description. The proposed association between asymmetric fetal loading and torsion, and the interpretation of Doppler indices as indicating uterine and fetal nonviability, rest on one clinical observation and cannot establish causality or generalisable diagnostic thresholds; case series or prospective studies are required for confirmation. Doppler ultrasonography is also operator-dependent and requires equipment capable of colour, power, and spectral modalities together with training in vascular waveform acquisition and interpretation; these requirements may limit its availability and reproducibility in general small animal practice relative to B-mode imaging alone. No formal comparison with normative feline uterine or ovarian Doppler reference ranges was possible, as such data have not, to our knowledge, been published for late-gestation queens.

In addition, resistive and pulsatility index values were assessed in real time using the ultrasound system’s automated calculations but were not archived; Doppler findings are consequently reported qualitatively, which precludes the comparison with quantitative reference data from other species and precludes retrospective statistical analysis.

## 5. Conclusions

This case report describes the successful emergency management of uterine torsion in a 4-year-old gravid queen by ovariohysterectomy, following a systematic pre-operative Doppler ultrasonographic assessment. Colour Doppler demonstrated absent arterial perfusion in the affected uterine segment, elevated vascular resistance indices in the ipsilateral uterine artery, and the complete absence of fetal umbilical flow and cardiac activity, providing direct pre-surgical evidence of irreversible vascular occlusion and fetal demise. These findings supported the decision to perform an en-bloc ovariohysterectomy without prior derotation. A serial haematological assessment documented severe acute regenerative anaemia and marked inflammatory leucocytosis, which improved progressively following the surgical removal of the necrotic uterus.

The intraoperative confirmation of asymmetric fetal loading—two kittens in the torted horn, fetal resorption in the contralateral horn—supports the hypothesis that unequal intraluminal mass distribution may constitute a biomechanical substrate for feline uterine torsion, corroborating the identical fetal distribution pattern reported by Ali et al. (2021) [14].

This report describes a systematic, multi-vessel Doppler protocol for feline uterine torsion, documenting both uterine arterial resistance indices and fetal cardiovascular status; given the sparse and likely incomplete feline literature discussed in Section 4.1, we make no claim beyond this. Because these observations derive from a single case, they are hypothesis-generating: Doppler ultrasonography may add information on tissue viability and fetal cardiovascular status beyond that available from B-mode imaging alone, but additional cases are needed before it can be recommended as a routine component of emergency assessment in gravid queens. Establishing normative Doppler reference values for the feline uterine and ovarian arteries in late gestation should be a priority for future investigation.

## Figures and Tables

**Table 1:** Haematological parameters of the queen at three time-points: on admission (Pre-op 1, 23 June 2026), the day before surgery (Pre-op 2, 24 June 2026), and the first post-operative morning (Post-op, 25 June 2026). All analyses were performed on the Mindray BC-60R Vet haematology analyser (Mindray Animal Care, Shenzhen, China). Reference intervals per manufacturer for the domestic cat.

Parameter	Unit	Pre-Op 1 23 June 2026	Pre-Op 2 24 June 2026	Post-Op 25 June 2026	Reference Interval
***White Blood Cell (WBC) Parameters***					
**WBC—Total leucocytes**	×10^9^/L	83.78 ^H^	58.49 ^H^	45.26 ^H^	3.46–17.50
**Neu#—Neutrophils**	×10^9^/L	64.51 ^H^	42.81 ^H^	28.38 ^H^	1.95–11.50
**Lym#—Lymphocytes**	×10^9^/L	12.73 ^H^	6.26	8.51 ^H^	0.73–7.40
**Mon#—Monocytes**	×10^9^/L	4.11 ^H^	4.97 ^H^	4.16 ^H^	0.06–0.98
**Eos#—Eosinophils**	×10^9^/L	1.76 ^H^	4.09 ^H^	4.07 ^H^	0.04–1.48
**Bas#—Basophils**	×10^9^/L	0.67 ^H^	0.35 ^H^	0.14	0.00–0.25
**Neu%—Neutrophil fraction**		0.770	0.732	0.627	0.300–0.835
**Lym%—Lymphocyte fraction**		0.162	0.107	0.188	0.070–0.600
**Mon%—Monocyte fraction**		0.049	0.085 ^H^	0.092 ^H^	0.008–0.080
**Eos%—Eosinophil fraction**		0.021	0.070	0.090	0.005–0.115
**Bas%—Basophil fraction**		0.008	0.006	0.003	0.000–0.023
***Red Blood Cell (RBC) Parameters***					
**RBC—Erythrocytes**	×10^12^/L	1.86 ^L^	1.76 ^L^	2.24 ^L^	6.30–11.82
**HGB—Haemoglobin**	g/L	29 ^L^	31 ^L^	37 ^L^	90–160
**HCT—Haematocrit**	L/L	0.088 ^L^	0.092 ^L^	0.125 ^L^	0.260–0.502
**MCV—Mean corpuscular vol.**	fL	47.2	52.2	55.7 ^H^	34.0–55.0
**MCH—Mean corp. Hb**	pg	15.6	17.6	16.4	11.0–18.0
**MCHC—Mean corp. Hb conc.**	g/L	330	337	296	285–384
**RDW-CV—RBC dist. width CV**		0.623 ^H^	0.598 ^H^	0.591 ^H^	0.142–0.266
**RDW-SD—RBC dist. width SD**	fL	87.1 ^H^	110.8 ^H^	114.8 ^H^	22.0–39.6
***Platelet (PLT) Parameters***					
**PLT—Platelets**	×10^9^/L	256	153 ^c^	412	140–595
**MPV—Mean platelet vol.**	fL	7.9 ^L^	8.8	9.2	8.6–18.4
**PDW—Platelet dist. width**		16.3	15.3	14.4	12.0–17.5
**PCT—Plateletcrit**	mL/L	2.01	1.35 ^L^	3.79	1.50–9.00
**IPF—Immature platelet fraction.**	%	9.5	9.1	15.5	0.7–28.0
***Reticulocyte (RET) Parameters***					
**RET#—Reticulocyte count**	×10^9^/L	140.2 ^H^	152.9 ^H^	164.2 ^H^	4.0–52.0
**RET%—Reticulocyte fraction**	%	7.54 ^H^	8.69 ^H^	7.33 ^H^	0.05–0.90
**IRF—Immature reticulocyte fraction.**	%	56.8 ^H^	66.7 ^H^	58.8 ^H^	0.0–33.0
**LFR—Low fluorescence ret.**	%	43.2 ^L^	33.3 ^L^	41.2 ^L^	66.0–100.0
**MFR—Mid fluorescence ret.**	%	29.8 ^H^	36.3 ^H^	35.0 ^H^	0.0–25.8
**HFR—High fluorescence ret.**	%	27.0 ^H^	30.4 ^H^	23.8 ^H^	0.0–8.5
**RHE—Ret. Hb equivalent**	pg	20.5	19.7	18.7	14.2–21.5

#: absolute count (×10^9^/L). %: relative fraction. ^H^: above upper reference limit. ^L^: below lower reference limit. ^c^: PLT Clump flag—analyser detected platelet aggregation; true platelet count may be higher. IRF: immature reticulocyte fraction; MFR: mid-fluorescence reticulocyte fraction; HFR: high-fluorescence reticulocyte fraction; IPF: immature platelet fraction; RDW: red cell distribution width.

**Table 2:** Summary of Doppler ultrasonographic findings by vessel.

Vessel	Side	Modality	Finding
Uterine artery, distal to torsion	Affected horn	Colour, spectral	Absent to markedly reduced arterial signal; turbulent/chaotic flow at the torsion site; markedly elevated-resistance waveform pattern with near-absent end-diastolic flow
Uterine artery	Contralateral horn	Colour	Partially preserved flow signal
Ovarian artery	Affected side	Spectral	Markedly elevated-resistance waveform, consistent with vascular compromise at the pedicle
Placental vasculature	Contralateral horn (resorption site)	Colour	Minimal, scattered vascular signal; formal spectral quantification not performed
Umbilical cord, both foetuses	Torted horn	Colour, spectral	Absent flow
Umbilical/ placental	Contralateral horn	Colour	No vascular signal identifiable

Resistive and pulsatility indices were calculated in real time to guide qualitative interpretation of flow impedance; numeric values were not archived and are not reported (Section 4.7, Limitations).

**Table 3:** Comparison of published feline uterine torsion case reports and the present case. NR: not reported; OHE: ovariohysterectomy; CS: caesarean section; DSH: domestic shorthair; POD: post-operative day; DIC: disseminated intravascular coagulation.

Reference	Breed; Age	Gravid Status/Stage	Horn/Torsion Degree	Fetal Outcome	Doppler Used	Maternal Outcome
Biller & Haibel, 1987 [1]	Domestic cat; NR	Late gestation	NR	All dead	No	Survived; OHE
Ridyard et al., 2000 [3]	Maine Coon; 15 months; 7.2 kg	Full-term (7 kittens)	NR; degree NR	All dead	No	Survived; OHE + intensive care; DIC suspected
Thilagar et al., 2005 [2]	Domestic cat; 2 years	Mid-gestation	Left horn; 180 deg at base + 360 deg at ovarian end	All dead (left horn)	No	Survived; OHE; recovery by day 5
Stanley & Pacchiana, 2008 [8]	Domestic cat; NR	Non-gravid (pyometra)	NR	N/A	No	Survived; OHE; metabolic abnormalities
De La Puerta et al., 2008 [9]	Domestic cat; NR	Non-gravid	NR	N/A	No	Survived; OHE
Kuroda et al., 2017 [4]	Maine Coon; 5 years	Full-term (day 60)	Right horn; 360 deg clockwise at the base	2 dead; 1 alive (left horn)—died POD 1	No	Survived; OHE + CS; blood transfusion
Tohumcu et al., 2021 [5]	Angora cat; NR	Late gestation	NR	NR	Yes (viability)	Survived; OHE
Ali et al., 2021 [14]	DSH; 2 years; pluriparous	Full-term (3 kittens)	Right horn; 720 deg	All dead: 2 (right) + 1 (left)	No	Survived; OHE after CS approach
Hendy & Elgohary, 2023 [6]	Shirazi cat; 7 years	Full-term (partial parturition preceded)	Left horn; 90 deg	All dead	No	Survived; OHE; fluid therapy only
Lalayev & Rahimli, 2024 [7]	Tabby cat; NR	~Day 45 gestation	Left horn; 270 deg	2 dead (torted horn)	Yes (viability)	Survived; OHE; discharged POD 1
Present case, 2026	Domestic cat; 4 years	Late gestation (2 kittens + 1 fetal resorption)	Right horn; 540 deg	All dead	Yes	Survived; OHE; full recovery

## Data Availability

The haematological data supporting the findings of this study are included in Table 1 of the manuscript. Original analyser printouts are available from the corresponding author upon reasonable request.

## Supplementary Materials

The following supporting information can be downloaded at: https://www.mdpi.com/article/10.3390/ani16172806/s1, Video S1: Intraoperative video of the torted right uterine horn as identified at laparotomy, prior to ligation and en-bloc resection; Figure S1: Close-up intraoperative photograph of the exteriorised torted uterine horn, showing the two kittens within, prior to resection; Figure S2: Intraoperative photograph of the ligated ovarian pedicles and uterine body prior to en-bloc resection; Figure S3: Photograph of the excised torted horn and contralateral horn immediately after resection; Figure S4: Photograph of the queen on the first post-operative morning, showing general condition and intravenous catheter placement; Figure S5: Photograph of the queen’s ventral abdomen on presentation, showing marked abdominal distension prior to surgery.

## Abbreviations

The following abbreviations are used in this manuscript:

B-mode	Brightness-mode ultrasonography
CBC	Complete blood count
CT	Computed tomography
DIC	Disseminated intravascular coagulation
EDV	End-diastolic velocity
ET-CO_2_	End-tidal carbon dioxide concentration
HCT	Haematocrit
HFR	High-fluorescence reticulocyte fraction
HGB	Haemoglobin
IPF	Immature platelet fraction
IRF	Immature reticulocyte fraction
K2EDTA	Dipotassium ethylenediaminetetraacetate
MCH	Mean corpuscular haemoglobin
MCHC	Mean corpuscular haemoglobin concentration
MCV	Mean corpuscular volume
MFR	Mid-fluorescence reticulocyte fraction
MPV	Mean platelet volume
NR	Not reported
OHE	Ovariohysterectomy
PI	Pulsatility index
PLT	Platelet count
POD	Post-operative day
PRF	Pulse repetition frequency
PSV	Peak systolic velocity
RBC	Erythrocyte count
RDW	Red cell distribution width
RET	Reticulocyte count
RI	Resistive index
SpO_2_	Peripheral oxygen saturation by pulse oximetry
USAMV	Universitatea de Stiinte Agronomice si Medicina Veterinara din Bucuresti (University of Agricultural Sciences and Veterinary Medicine of Bucharest)
UT	Uterine torsion
WBC	Total leucocyte count
